# Hyperthermia and Tumor Immunity

**DOI:** 10.3390/cancers13112507

**Published:** 2021-05-21

**Authors:** Ather Adnan, Nina M. Muñoz, Punit Prakash, Peiman Habibollahi, Erik N. K. Cressman, Rahul A. Sheth

**Affiliations:** 1 Texas A&M Health Science Center, Texas A&M College of Medicine, Houston, TX 77030, USA; aadnan@exchange.tamu.edu; 2 Department of Interventional Radiology, The University of Texas MD Anderson Cancer Center, Houston, TX 77030, USA; NMMunoz@mdanderson.org (N.M.M.); PHabibollahi@mdanderson.org (P.H.); ECressman@mdanderson.org (E.N.K.C.); 3 Department of Electrical and Computer Engineering, Kansas State University, Manhattan, KS 66506, USA; prakashp@ksu.edu

**Keywords:** hyperthermia, ablation, immunotherapy, cancer

## Abstract

**Simple Summary:**

Minimally invasive procedures that directly deliver heat into tumors are commonly used to treat a wide range of malignancies. In this review, we discuss the potential role that these procedures can play in not only destroying small tumors but also educating the immune system to recognize and attack tumors throughout the body.

**Abstract:**

Thermal ablation is a cornerstone in the management of cancer patients. Typically, ablation procedures are performed for patients with a solitary or oligometastatic disease with the intention of eradicating all sites of the disease. Ablation has traditionally played a less prominent role for patients with a widely metastatic disease. For such patients, attempting to treat numerous sites of disease compounds potential risks without a clear clinical benefit and, as such, a compelling justification for performing an intervention that is unlikely to alter a patient’s clinical trajectory is uncommon. However, the discovery of immune checkpoints and the development of immune checkpoint inhibitors have brought a new perspective to the relevance of local cancer therapies such as ablation for patients with a metastatic disease. It is becoming increasingly apparent that local cancer therapies can have systemic immune effects. Thus, in the new perspective of cancer care centered upon immunologic principles, there is a strong interest in exploring the utility of ablation for patients with a metastatic disease for its immunologic implications. In this review, we summarize the unmet clinical need for adjuvant interventions such as ablation to broaden the impact of systemic immunotherapies. We additionally highlight the extant preclinical and clinical data for the immunogenicity of common thermal ablation modalities.

## 1. Introduction

Thermal ablation is a cornerstone in the management of cancer patients [1]. Both heat- and cold- based technologies have been incorporated into contemporary treatment paradigms for various malignancies. Typically, ablation procedures are performed for patients with solitary or oligometastatic cancers with the intention of eradicating all sites of the disease. These minimally invasive approaches have substantially broadened the modern cancer physician’s capabilities for controlling the tumor burden in patients with a solitary or oligometastatic disease. However, ablation plays a less prominent role in the large proportion of patients with a widely metastatic disease. For such patients, attempting to treat numerous sites of the disease compounds the potential risks without a clear clinical benefit and, as such, a compelling justification for performing an intervention that is unlikely to alter a patient’s clinical trajectory is uncommon. As conventional wisdom states, systemic diseases require systemic therapies. 

Over the past decade, however, this conventional wisdom has begun to evolve due to the recent renaissance in cancer immunotherapies. The discovery of immune checkpoints and the development of immune checkpoint inhibitors has revolutionized treatment algorithms across the cancer spectrum [2]. These advances in systemic therapies have also brought a new perspective to the relevance of local cancer therapies such as ablation for patients with a metastatic disease. It is becoming increasingly apparent that local cancer therapies can have systemic immune effects. Thus, in the new perspective of cancer care centered upon immunologic principles, there is a strong interest in exploring the utility of ablation for patients with a metastatic disease for its immunologic implications [3,4,5,6,7]. In this review, we summarize the unmet clinical need for adjuvant interventions such as ablation to broaden the impact of systemic immunotherapies. We additionally highlight the extant preclinical and clinical data for the immunogenicity of common thermal ablation modalities.

## 2. Landscape of Systemic Immunotherapies and the Need for Adjuvant Interventions Seen through the Lens of Hepatocellular Carcinoma

Immunotherapy represents a major disruptive innovation in cancer care. While immunotherapies such as systemic cytokines have been used clinically for decades, the field has recently undergone a revolution with the advent of immune checkpoint inhibitors. Immune checkpoint blockade antibodies that augment the effector T cell activity by blocking inhibitory molecules such as programmed cell death 1 (PD-1), its ligand PD-L1 and cytotoxic T-lymphocyte antigen-4 (CTLA-4) have achieved unprecedented advances in numerous cancer types [2]. The outcomes from immunotherapy are remarkable not only for the fact that they can occur in patients refractory to all other lines of cancer therapy but also for their durability [8]; patients with an advanced malignancy have exhibited complete remissions lasting over a decade following treatment with immunotherapy.

Despite paradigm-shifting advances in several malignancies, current immunotherapy approaches are limited in several ways. These limitations are well-exemplified in the case of a hepatocellular carcinoma (HCC), a malignancy that is commonly treated with ablation. An HCC is the third most common cause of cancer-related deaths globally and in the United States it is the fastest growing cause of cancer-related deaths [9]. For patients with small, solitary HCC lesions, local tumor control with hyperthermia modalities is highly effective and is the standard of care. However, the vast majority of patients with an HCC present with an advanced stage disease, a diagnosis with few treatment options. The intrinsic tumor resistance and underlying liver dysfunction limit the efficacy and safety of systemic chemotherapies [10]. There is an urgent clinical need for new systemic therapies for an HCC.

There is a strong rationale for immunotherapy in an HCC. Patients with HCC tumors expressing high levels of PD-L1 have a significantly poorer prognosis than patients with a lower expression and the tumor expression of PD-L1 is an independent predictor for post-operative recurrence in patients with an HCC [11]. Phase I/II trials of anti-PD-1 and anti-CTLA-4 checkpoint inhibitors for HCCs have demonstrated durable responses; however, such responses only occur in a minority of patients [12]. For example, the CheckMate 040 trial, a phase I/II study of an anti-PD-1 checkpoint inhibitor for patients with an advanced HCC, demonstrated an objective response rate of only 20% in the dose-expansion cohort [13]. As a result, there is no immunotherapy approved as a monotherapy for first-line use in an HCC.

Beyond the intrinsic immunosuppressive nature of an HCC, the liver itself can serve as a “trap” for tumor-specific T cells. Yu et al. demonstrated in multiple mouse models as well as in patients that liver tumors sequester T cells from the circulation leading to T cell killing in a macrophage-driven mechanism. This diminishes the efficacy of immunotherapy not only within the liver lesion but also for other tumors across the body. The study also found that patients with liver metastases have diminished circulating T cells compared with those patients who do not have [14] liver metastases.

A major question in immunotherapy, therefore, is how to broaden treatment responses to a greater proportion of HCC patients. The answer lies in combination regimens that modify the tumor microenvironment to potentiate the immunotherapy drug as recently demonstrated by the IMbrave150 trial that combined an anti-PD-L1 antibody with an anti-angiogenesis antibody [15]. It is believed that the T cell recognition of neoantigens contributes to the efficacy of checkpoint inhibitors [16]. These neoantigens, however, are shielded from antigen-presenting cells (APCs) by an immunosuppressive microenvironment. The microenvironment enables tumors to escape host immunosurveillance by multiple mechanisms including the recruitment of inhibitory cells, the modulation of APCs toward immunosuppressive phenotypes and the inhibition of the antigen presentation functionality of APCs [2].

The identification of resistance mechanisms to systemic immunotherapy for HCCs has been replicated for multiple other cancer types. Unfortunately, the objective response rates to checkpoint inhibitor therapy remain below 50% across all malignancies [17]. Thus, without an adjuvant method to alter the immunosuppressive microenvironment and drive the adaptive immune activation, immunotherapy is unlikely to improve outcomes for the majority of cancer patients.

## 3. Immunogenicity of Ablation: It Starts with Immunogenic Cell Death

An understanding of the mechanisms of systemic immunotherapy resistance requires an appreciation for the necessary steps to activate the adaptive immune response. It has long been appreciated that not all forms of cancer cell death are equal. Several forms of cell death initiate the immunogenic cascade of APC activation and T cell stimulation, clonal expansion and tumor eradication; such forms of cell death are termed immunogenic cell death (ICD). However, other forms of regulated cancer cell death can lead to T cell anergy and immune tolerance, thus inhibiting an anti-tumor immune response. Therefore, there is great interest in understanding the various forms of cell death that occur following local cancer therapies and prioritizing those modalities that result in ICD over other forms of regulated cell death; combining such modalities with systemic immunotherapies has the potential to greatly broaden the applicability of immunotherapy regimens for cancer patients.

A recently published consensus guideline [18] detailed the requisite features that must be present for ICD to occur. The establishment of anti-tumor adaptive immunity depends upon two characteristics of the manner in which cancer cells die: antigenicity and adjuvanticity. The former refers to the release of antigens upon a cell’s death, which naïve T cell clones can recognize. Whilst the vast majority of a cancer cell’s antigenic epitopes are identical to self-antigens and are therefore non-immunogenic, mutated proteins unique to the malignant tissue lead to neoepitopes that can activate quiescent T cells. Adjuvanticity refers to the fact that the antigen release by cancer cells and the subsequent expression by APCs is necessary but alone is insufficient to drive a potent immune response. There must also be the release of “danger signals” commonly known as damage-associated molecular patterns (DAMPs) concurrent with the antigen release to recruit and activate APCs. While many DAMPs such as extracellular DNA, uric acid and heat shock proteins are commonly present in almost every cell type, the timing and method in which they are released plays a key role in whether APC activation occurs or not. Thus, although any given local cancer treatment modality may lead to cancer cell death, this does not guarantee that ICD will occur. The following sections briefly review the mechanisms of immunogenic cancer cell death with a special consideration to hyperthermia.

### 3.1. Hyperthermia-Induced Antigenicity

Whereas antigenicity classically arises from highly immunogenic microbial proteins or microbe-associated molecular proteins (MAMPs) not covered by central tolerance or exceptionally in healthy cells via stress-induced post-translational modifications or the activation of human endogenous retroviruses, cancer cells generate immunogenic antigens largely due to their high rate of mutation [18]. Two major classes of antigens related to tumors have been described. The first are tumor neoantigens (TNAs), which can arise from non-synonymous mutations in coded regions of the genome that result in the synthesis of a new peptide that is ultimately expressed on the MHC complex [19]. TNAs may also arise from post-translational modifications perhaps induced by stressful conditions of the tumor microenvironment [20]. The second are tumor-associated antigens (TAAs), which are non-mutated self-antigens to which T cell tolerance is incomplete. TNAs are hypothesized to play the predominant role in cancer cell antigenicity because of the lack of influence by the central tolerance [19].

Several factors contribute to the successful generation of an immunogenic antigen such that only a fraction of mutations result in one. For example, the mutational burden of a tumor, which is heterogenous between patients and even within a patient across space and time [21,22], plays a role in the probability of a generation of a TNA [23,24]. Perhaps more relevant to ICD-inducing cancer therapies such as ablation is the ability to perform antigen presentation. Cancer cells are prone to multiple defects in this process including the preferential expansion of subclones that do not express immunogenic epitopes [25] or impairments in key components of the antigen-presenting machinery [26,27]. On top of this, normal processing already limits the frequency of the presentation of peptides generated by the proteasome (estimated at <0.1%) [28] with a preference for those actively undergoing a rapid synthesis [29]. Thus, even if a potentially immunogenic antigen is present within a cancer cell, it may never result in an immune response for multiple reasons.

Ablation is advantageous as a cancer therapy in this regard as it causes cell death and the release of antigens perhaps otherwise not adequately presented. Additionally, such extracellular antigens can be sampled by dendritic cells and presented on MHC class II complexes [30]. While the MHC class I-restricted antigen presentation to CD8+ T cells is thought to primarily mediate the anti-tumor immune response, and indeed there is substantial evidence that ablation induces cytotoxic T lymphocytes (reviewed in detail in subsequent sections), there is a growing appreciation for the role of CD4+ T cell-mediated anti-tumor immunity [31,32,33]. Furthermore, there is evidence that hyperthermia can mitigate the DNA repair system and may be able to potentiate DNA-targeting anti-cancer therapies or itself promote the generation of tumor neoantigens [34]. Finally, as alluded to earlier, another theoretical avenue by which hyperthermia can induce antigenicity in cancer cells is the modulation of the tumor microenvironment to induce the various stress-related production of neoantigens by such processes as protein oxidation or the activation of endogenous retroviruses although the significance and exploitation of this mechanism in the context of ablation remains largely unexplored.

### 3.2. Hyperthermia-Induced Adjuvanticity

Comprehensive reviews of DAMPs mechanistically linked to cancer have been reported elsewhere [18,35]. Briefly, the most prominent players include nucleic acids such as mitochondrial DNA and self-RNA, which induce the production of immunostimulatory type I IFNs; extracellularly released ATP, a ‘find me’ signal potentiating the recruitment of dendritic cells; the surface expression of calreticulin, heat shock proteins and other ER chaperones, which collectively serve as ‘eat me’ signals facilitating the phagocytic uptake of tumor cells; and HMGB1, whose role is controversial but may stimulate the efficient processing and cross-presentation in APCs [18,36]. Many of these arise as a consequence of the failure of cytoprotective processes such as autophagy (e.g., ATP) and the phosphorylation of eukaryotic translation initiation factor 2 subunit alpha (e.g., calreticulin and heat shock proteins) as part of the ‘integrated stress response’ [37].

There is considerable evidence that hyperthermia potentiates the adjuvanticity of tumor cell immunogenicity; for example, by the release of many of the aforementioned DAMPs (reviewed in detail in subsequent sections). Several of the immunostimulatory effects of hyperthermia on the tumor microenvironment include an increased tumor perfusion and reoxygenization allowing for an enhanced immune-effector uptake and a decreased hypoxia-related immunosuppression, an increased immune cell trafficking and effector function and increased pro-inflammatory cytokines [38,39]. Furthermore, we have demonstrated that hyperthermia can modulate the myeloid population toward an M1 phenotype within the tumor microenvironment by altering the tumor-intrinsic *Wnt*-β-catenin pathway [40].

## 4. Evidence for the Immunogenicity of Ablation

**Figure 1:**
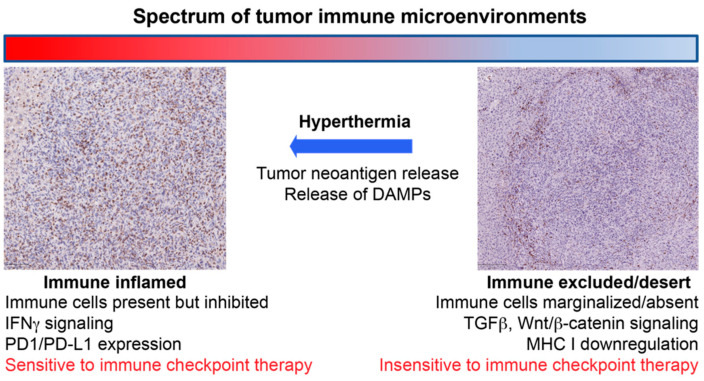
Schematic illustrating the concept of how hyperthermia can convert immunosuppressive tumor immune microenvironments to “inflamed” microenvironments that are sensitive to an immune checkpoint inhibitor therapy.

The generation of ICD has been identified in multiple locoregional cancer therapies including radiation therapy [18], direct intratumoral immunotherapies [41,42,43] and hyperthermia [40]. One potential advantage of hyperthermia over radiation is the absence of lymphopenia that can follow radiation therapy [44,45,46]. With regard to hyperthermia, there are numerous modalities in clinical practice [47] and in development [48] that can generate heat within tumoral tissues. However, as with radiation therapy [49,50], the manner in which thermal energy is deposited within the tissues undoubtedly plays a pivotal role in the antigenicity and adjuvanticity of the resultant form of cell death that follows. In the subsequent sections, we review the existing literature for the most common hyperthermia modalities with an emphasis on their potential for immune stimulation (Figure 1).

### 4.1. Radiofrequency Ablation

Radiofrequency ablation (RFA) has been used in the clinical arena for decades; accordingly, the preponderance of evidence regarding immune activation following hyperthermia can be found in the RFA literature. In the preclinical setting, multiple studies have demonstrated that RFA releases numerous DAMPs including RNA, DNA, heat shock proteins and uric acid; furthermore, there is an elaboration of immunogenic cytokines including interleukin-1b (IL-1b), IL-6 and IL-8 [51,52,53,54]. As a consequence of these immunogenic alterations to the tumor immune microenvironment, RFA has also been shown to reduce the levels of regulatory T cells [53] and studies have also demonstrated an increase in the presence of tumor-specific T cells [55,56] following RFA. Furthermore, this increase in T cells was associated with an improvement in survival. For example, Hiroishi et al. [56] reviewed 20 patients with an HCC treated with RFA or chemo-embolization. They measured the levels of peripheral CD8+ T cells with T cell receptors targeting common tumor-associated antigens found in an HCC prior to and following treatment. They found that the majority of patients (80%) exhibited an increase in circulating tumor-specific T cells following locoregional therapy and that this increase in T cells was significantly associated with a progression free survival. Likewise, Widenmeyer et al. [57] studied 49 patients with primary or secondary liver tumors treated with RFA and found that in a small number of patients, circulating T cells targeting tumor-specific antigens could be seen following, but not prior to, RFA.

Evidence of systemic tumor immunity following RFA has also been identified in animal studies. Dromi et al. [58] evaluated the immunologic impact of a partial RFA in a heterotopic urothelial carcinoma mouse model. A partial RFA was performed to ensure that viable tumor antigens remained to allow for the subsequent APC presentation and activation; this approach also allowed for the evaluation of both local and systemic tumor immunity. Following RFA, the animals were rechallenged with a repeat injection of the same tumor cell line in the contralateral flank to evaluate for immunologic “memory”. RFA was also combined with the intratumoral delivery of dendritic cells to promote the antigen presentation. Both the monotherapy and combination therapy arms demonstrated an inhibition in rechallenge experiments, highlighting one of the hallmarks of adaptive immunity.

Contrasting the evidence of increased anti-tumor immunity following RFA is the identification of increased immunosuppressive markers. For example, we [40] and others [59,60,61,62,63] have shown that RFA results in an increased expression of multiple immune checkpoints including PD-1, PD-L1, CTLA-4 and VISTA. This finding is generally interpreted as deleterious to adaptive immune activation and certainly such inhibitory pathways can effectively abrogate meaningful cytotoxic T cell activity. However, these findings can also be interpreted as an expected response of the immune homeostasis machinery. The activation of effector T cells by any method is typically accompanied by counterbalancing mechanisms such as an immune checkpoint expression to prevent unopposed activation and excessive cytotoxicity; thus, in several ways, the increase in the checkpoint expression after RFA can be seen as a reflection of effective, albeit limited, T cell activation. Furthermore, many of these checkpoints can be targeted with systemic therapies such as checkpoint inhibitors, thus potentiating a sustained anti-tumor immune response after RFA.

Such combination strategies have been explored in both the preclinical and clinical setting. In a retrospective study, Shi et al. [63] reviewed the influence of RFA of colorectal liver metastases on immune infiltration in the primary tumor for patients who underwent primary resection following liver RFA. Ablation of the liver metastases significantly increased the T cell infiltration in the primary tumor but they also observed an increase in the PD-L1 expression by the tumor cells. They then took this observation back to the preclinical setting and combined RFA with checkpoint inhibitor therapy in a mouse model of colorectal cancer and found that the combination arm exhibited improved T cell responses and overall survival.

Combining RFA with checkpoint inhibition has also been evaluated in a prospective clinical trial. In a single arm study, 32 patients with an HCC were treated with the CTLA-4 inhibitor tremelimumab and also underwent a subtotal RFA of the liver tumor [64]. No dose-limiting toxicities were identified, an important finding particularly given the toxicity profile of a few CTLA-4 inhibitors. Biopsies also indicated an increase in CD8+ T cells within the tumor although, as a single arm study, the relative contribution of the checkpoint inhibitor therapy versus the RFA procedure was not known.

### 4.2. Microwave Ablation

Microwave ablation (MWA) typically results in larger ablation zones in a shorter amount of time compared with RFA and, as such, has largely replaced RFA as the preferred thermal ablation modality at many institutions. Unfortunately, however, there are limited data on the immunologic ramifications of MWA and, as discussed previously, it cannot be assumed that RFA and MWA are interchangeable from an immunologic perspective.

Studies investigating the immune response following MWA report an increase in circulating immune cells and pro-inflammatory cytokines. In a clinical study of 89 patients, Dong et al. [65] reviewed pathologic findings from biopsy samples following MWA of HCC tumors. They found a significant increase in multiple immune cell types following MWA including T cells, NK cells and macrophages. In another clinical study of 45 patients with an HCC, Zhang et al. [66] measured the T cell subsets and cytokines from peripheral blood before and after treatment with MWA. They reported a significant increase in CD3+ cells, CD4+ cells and IL-12 one month after treatment as well as a decrease in IL-4 and IL-10. A similar study by Zhou et al. [67] enrolled 30 patients with an HCC and measured the T cell subsets from peripheral blood before and after treatment with MWA. They found relatively stable concentrations of CD3+ and CD4+ cells but an increase in Th17 cells 24 h after treatment. Additionally, a high baseline concentration of circulating Th17 cells, instead of a transient elevation induced by MWA, was a risk factor for a tumor recurrence. In a preclinical study of osteosarcoma in mice, rats and a human cell line, Yu et al. [68] found that MWA elicited ICD (evidenced by the expression or release of calreticulin, HMGB1 and ATP) and an increase in CD8+ T cells. Furthermore, mice and rats were protected from a lethal challenge with tumor cells after vaccination with ablated tumor cells plus a supernatant; this protection was lost partially after CD4+ depletion but completely after CD8+ cell depletion, indicating that CD8+ T cells may be responsible for MWA-induced immunoprotection.

Data on the influence of MWA protocol parameters on the immune response are limited. The aforementioned study by Yu et al. [68] compared MWA of 10, 20 and 30 min duration, concluding that 20 min was the ‘sweet spot’ sublethal dose that maximized the expression of ICD-associated markers while minimizing non-immunogenic cell lysis. One preclinical study by Velez et al. [60] compared pro-inflammatory signals and extrahepatic tumor growth in mice with breast adenocarcinoma treated with either a slower lower-power (5 W for 120 s) MWA, a faster higher-power (20 W × 15 s) MWA, RFA or a sham liver ablation. They found increased concentrations of liver HSP 70 and macrophages in the periablational rim following RFA and lower-power 5 W MWA only as well as elevations in IL-6, VEGF and HGF following 5 W MWA and RFA compared with 20 W MWA and the control group. They also found an increased extrahepatic tumor growth following 5 W MWA and RFA, concluding that a faster heating MWA protocol may mitigate part of the post-ablation inflammatory milieu that contributes to distant pro-oncogenic effects. A clinical study by Zhao et al. [69] of 43 patients with hepatic malignancies compared levels of serum cytokines before and after MWA. Significant elevations greater than twofold were found in levels of IL-2, IL-1β, IL-6, IL-8, IL-10 and TNF-α after MWA. Elevations in IL-2 and IL-6 were positively correlated with the energy output.

There is preclinical and clinical evidence to support the use of MWA combined with immunotherapy. Jing et al. reported two preclinical studies in mice with hepatic tumors evaluating dendritic cell immunotherapy in combination with MWA. The first study [70] included mice injected with an HCC cell line and utilized dendritic cells primed to the lysate of the same cell line. The experiment arms included MWA only, dendritic cells only or both. The levels of regulatory T cells and Th17 cells were lower following a combination therapy rather than either therapy alone. Similarly, when rechallenged in the contralateral flank with the same cell line, the combination therapy group showed an earlier tendency to suppress tumor growth compared with MWA alone. The second study [71] was designed similarly but included a dendritic cell-derived exosome group. They found no significant differences between MWA plus dendritic cells or MWA plus dendritic cell-derived exosomes: both groups resulted in higher levels of CD8+ T cells and IFN-γ and lower levels of Treg cells and IL-10 compared with MWA alone. Studies evaluating MWA combined with immune checkpoint inhibitors similarly show improved efficacy. In a mouse model of breast cancer, Zhu et al. [72] compared a treatment with an MWA versus PD-1 and CTLA-4 blockade versus an MWA plus PD-1 and CTLA-4 blockade versus no treatment. Compared with either single therapy, the combination group significantly prolonged tumor-bearing mouse survival, protected most mice from a tumor rechallenge and further augmented increases in local and systemic CD8+ T cells and in plasma IFN-γ. In a clinical trial designed similarly to the Duffy et al. [64] study, Xie et al. [73] evaluated the combination of the CTLA-4 inhibitor tremelimumab with a partial MWA in patients with biliary tract cancer. No dose-limiting toxicities were found in this study. Immune profiling revealed an increase in circulating activated T cells.

### 4.3. Focused Ultrasound

High intensity focused ultrasound (HIFU) differentiates itself from other hyperthermia modalities by the fact that it is completely non-invasive. Furthermore, in addition to hyperthermia, HIFU can also generate non-thermal mechanical damage within the tissue through a phenomenon known as acoustic cavitation. From an immunologic perspective, this mechanism of action should be very effective at releasing DAMPs and this hypothesis has been borne out in both clinical and preclinical studies.

Numerous studies suggest that HIFU causes ICD in cancer cells and the subsequent release of danger signals including HSP-27, HSP-60, HSP-70, HSP-72, HSP-73, tumor-associated antigens and ATP [74,75,76,77,78,79]. For example, in an in vitro study of a murine colon adenocarcinoma cell line, Hu et al. [76] found an increased release of endogenous danger signals (HSP60 and ATP) following treatment with HIFU. One clinical study of biopsies obtained from breast cancer patients by Wu et al. [78] similarly found increased HSPs following HIFU. Additionally, newly expressed tumor antigens and danger signals are able to persist after thermal ablation supporting the notion that they can be used to activate the host immunity and elicit an immune response against the tumor.

Indeed, subsequent studies showed evidence of HIFU-induced immunological anti-tumor effects such as an increased immune cell infiltration and the ability of tumor debris to activate immune cells. The aforementioned study by Hu et al. [76] found that exposing dendritic cells to the supernatant of the HIFU-treated tumor cells caused an increased expression of co-stimulatory molecules on dendritic cells and the enhanced secretion of TNF-a and IL-12 by macrophages and dendritic cells. In a mouse model of an HCC, Zhang et al. [80] found that the tumor lysate following HIFU of HCC tumors provided immunity against a tumor challenge when used to immunize mice against the tumor cell line. Similarly designed studies by the same group [81,82] also found an increased number of mature dendritic cells and splenic lymphocyte proliferation following co-incubation with HIFU-ablated tumor debris as well as an enhanced cytotoxicity and number of CD8+ T cells following HIFU treatment. In a clinical study, Xu et al. [83] evaluated the influence of HIFU in patients with breast cancer. The tumors treated with HIFU prior to a surgical resection were found to have APCs localized along the periphery of the ablation margin and a significant increase in dendritic cells, macrophages and B lymphocytes compared with tumors who underwent a surgical resection alone. Another clinical study by Lu et al. [84] similarly reported an increased tumor margin infiltration by CD3, CD4, CD8, CD4/CD8 and B lymphocytes and NK cells in patients with HIFU-treated breast cancer. The inhibition of the immunosuppressive effects of miR-134 on CD86 is a potential mechanism contributing to the immunological anti-tumor effects of HIFU [85].

Strategies to optimize the delivery of thermal FUS and its immunostimulatory effects have been pursued. A study by Liu et al. [86] investigated whether the use of a sparse-scan strategy to create multiple non-overlapping thermal ablations, effectively increasing the surface area of the periablation region, could enhance the anti-tumor response induced by HIFU. A sparse-scan HIFU was shown to be more effective than a dense-scan HIFU in enhancing the infiltration of dendritic cells into tumor tissues and promoting their maturation in situ. Bandyopadhyay et al. [87] showed that a non-ablative, low-intensity FUS (LOFU) protocol compared with HIFU may be more effective in increasing the immunogenic presentation of tumor antigens and resulting in the reversal of tumor-induced T cell tolerance.

It is hypothesized that HIFU-induced ablation alone is insufficient to cause a lasting immune response against the tumor particularly those prone to a recurrence or metastasis in part because the resultant coagulative necrosis dampens the immunostimulatory response [88]. Contemporary studies have investigated the therapeutic potential of combined HIFU and immunotherapy with promising preclinical results [89,90,91,92,93]. For example, Ran et al. [89] performed an adoptive transfer of splenic T lymphocytes from mice with an HCC treated with HIFU into new mice with an HCC. The adoptive transfer of HIFU-activated T lymphocytes significantly increased tumor-infiltrating T lymphocytes, IFN-γ-secreting cells and survival time in tumor-bearing mice and inhibited the tumor growth and progression. A study by Silvestrini et al. [90] evaluated the importance of timing the immunotherapy in relation to the HIFU in murine breast cancer. Compared with the treatment with immunotherapy (CpG + PD-1 blockade) alone, the treatment with coincident ablation and immunotherapy led to diminished abscopal effects. However, priming with immunotherapy for 1 week prior to ablation rescued the therapeutic effect and led to a decrease in the macrophages and MDSCs and enhanced IFN-γ-producing CD8+ T cells and the M1 macrophage fraction. A continued combined treatment led to a higher complete response rate. After a tumor rechallenge followed by a repeat treatment, survival was improved with primed ablation versus immunotherapy alone. Two subsequent studies by the same group offered insights into the mechanisms contributing to these findings. The first study by Chavez et al. [91] found an enhanced systemic tumor antigen cross-presentation, type I IFN release from tumor cells, transcription of genes related to T cell activation and a CD169+ subpopulation of macrophages and dendritic cells in the combined therapy group. The second study by Fite et al. [93] found an increase in the expression of pattern recognition receptors across multiple families only in the local tumor with HIFU alone, which was further increased with the addition of immunotherapy both locally and in distant tumors. Additionally, the HIFU-induced upregulation of the genes *Il6* and *Il1β,* which the authors hypothesized could promote a chronic inflammatory pro-tumor environment, was attenuated and the tumor infiltration by dendritic cells was enhanced with the addition of immunotherapy. Taken together, these findings suggest that HIFU primed with immunotherapy may elicit a more robust, global and lasting anti-tumor adaptive immune response compared with either monotherapy.

## 5. Future Directions

The clinical unmet need for an adjuvant intervention to boost the efficacy of systemic immunotherapies coupled with the immunologic impact of ablation has led to the recent activation of numerous combination clinical trials (Table 1).

These trials will provide crucial advances in our knowledge of both the barriers to immunotherapy and the efficacy of ablation to overcome them. They will also help to address key questions that remain unanswered including such fundamental questions as (1) which modality is the most effective from an immunologic perspective, (2) how should ablation and immunotherapy be sequenced and (3) what are the ideal properties of target lesions or lesions that should be treated with ablation?

In one light, the diversity of thermal ablation modalities and procedural techniques is a boon for immuno-oncologists as a great range of variables can be adjusted to identify synergies with systemic immunotherapies. From a practical perspective, however, it is unrealistic to perform optimizations of these parameters in a clinical trial setting and so it is possible that clinical trials may be unsuccessful not because of a fundamental flaw in the rationale for combining hyperthermia with immunotherapy but because of assumptions that had to be made for the sake of expediency in the study design. Such challenges can be overcome by a rigorous investigation of the mechanistic framework underpinning the immunologic ramifications of hyperthermia in the preclinical setting. While most prior preclinical studies have focused on the effects of hyperthermia (e.g., its influence on immune cell infiltration and changes in cytokine expression), few have delved deeper into the underlying mechanisms. An appreciation for the cellular and molecular perturbations caused by hyperthermia on the tumor microenvironment will be invaluable in designing strategies to maximize the immune effects of hyperthermia.

## 6. Conclusions

There is a substantial unmet need for adjuvant interventions to augment the efficacy of a systemic immune checkpoint inhibitor therapy. Numerous barriers to immunotherapies exist including the elaboration of immunosuppressive cytokines, immunosuppressive myeloid cells within the tumor microenvironment and a lack of exposure of tumor antigens to APCs. Hyperthermia modalities have demonstrated significant promise for addressing these barriers in preclinical models and multiple ongoing clinical trials will evaluate their efficacy in patients. As our mechanistic understanding of the immunologic ramifications for these interventions grows, we will be able to tailor our approaches in a patient-specific manner.

## Figures and Tables

**Table 1:** Examples of ongoing clinical studies combining ablation with immunotherapies.

Trial	Phase	Disease	Ablation Modality	Immunomodulator	Endpoints
NCT02833233	Pilot	Breast cancer	Cryoablation	Anti-PD-1 antibody plus anti-CTLA-4 antibody	Safety
NCT02821754	I/II	HCC, biliary tract tumor	RFA or cryoablation	Anti-PD-1 antibody, anti-CTLA-4 antibody	Safety, PFS
NCT02626230	Pilot	RCC	Cryoablation	Anti-CTLA-4 antibody	Safety, RR
NCT02559024	I	Colorectal cancer	RFA	Anti-OX40 antibody	Safety, immune response
NCT02469701	II	NSCLC	Cryoablation	Anti-PD-1 antibody	RR
NCT02437071	II	Colorectal cancer	RFA	Anti-PD-1 antibody	Safety, RR
NCT02423928	I	Prostate cancer	Cryoablation	DCs, cyclophosphamide, anti-CTLA-4 antibody	Safety
NCT02311582	I/II	Malignant glioma	Laser ablation	Anti-PD-1 antibody	Safety, PFS, OS
NCT02250014	I	Prostate cancer	Cryoablation	GM-CSF	Immune response, PSA level
NCT01853618	I	HCC, biliary tract tumor	RFA or cryoablation	Anti-CTLA-4 antibody	Safety, feasibility, RR, TTP, OS
NCT03237572	Pilot	Breast cancer	HIFU	Anti-PD-1 antibody	Immune response, safety

RCC = renal cell carcinoma; NSCLC = non-small cell lung cancer; PFS = progression free survival; RR = response rate; OS = overall survival; TTP = time to progression; PSA = prostate-specific antigen.
